# Internal Endpoint Cooking Temperature Alters Quality and Consumer Acceptability of Pork Loin Chops

**DOI:** 10.3390/foods14122052

**Published:** 2025-06-11

**Authors:** Savannah L. Douglas, Ricardo J. Barrazueta-Coredero, Gabriela M. Bernardez-Morales, Nina E. Gilmore, Linda S. Barahona-Dominguez, Sungeun Cho, Jason T. Sawyer

**Affiliations:** 1 Department of Animal Sciences, Auburn University, Auburn, AL 36849, USA; sld0060@auburn.edu (S.L.D.); rzb0143@auburn.edu (R.J.B.-C.); gzb0063@auburn.edu (G.M.B.-M.); neg0027@auburn.edu (N.E.G.); 2 Department of Poultry Sciences, Auburn University, Auburn, AL 36849, USA; lsb0042@auburn.edu (L.S.B.-D.); szc0181@auburn.edu (S.C.)

**Keywords:** cooked color, electronic nose, pork chops, sensory analysis, texture analysis

## Abstract

Quality and consumer satisfaction of meat products could be influenced by endpoint cooking temperatures. Attributes of pork, such as palatability, cooking loss, and internal color, influence consumer acceptability. The degree of doneness was evaluated on pork chop characteristics of texture, cooking loss, consumer acceptability, and electronic nose. Pork loin chops (N = 264) were allocated randomly to one of three endpoint degrees of doneness (63 °C, 71 °C, and 79 °C). Cooking pork chops to an internal temperature of 79 °C caused the cooked color to be darker (*p* < 0.0001) and less red (*p* = 0.0057). In addition, chops cooked to a 63 °C degree of doneness had greater moisture and lower shear force values (*p* < 0.0001). Consumer panel ratings of flavor profiles were greater for juiciness, texture, and tenderness (*p* < 0.0001) when chops were cooked to a 63 °C degree of doneness. Electronic nose analysis of the changes in cooked volatiles can impact the overall flavor and aroma profiles of pork loin chops. These findings conclude that cooking pork chops to an internal temperature of 63 °C improves the overall eating quality, acceptability, and cooking characteristics of pork loin chops. However, more information on the use of an optimal endpoint cooking temperature is needed to improve consumer awareness of pork chop quality.

## 1. Introduction

Pork is a global product widely consumed, comprising 36% of total meat consumption [1,2]. It is well known throughout the literature that consumers will choose proteins at the retail level based on price, packaging, color, and flavor. Pork products remain valued because of their versatility, texture, flavor, and affordability [3]. Furthermore, providing wholesome and safe pork is essential for the meat industry to achieve consumer satisfaction [4]. Changes to cooked proteins such as pork have been well documented and describe altered characteristics such as degree of doneness, loss of moisture, and reduced fat, often impacted at the muscle fiber level. It has been reported that pork flavor, tenderness, and texture will affect the consumers’ overall quality perception of a product cut [5]. Cooked protein quality is highly influenced by the internal endpoint temperature (degree of doneness) in which the product is cooked [6]. Cooking temperatures can alter the amount of moisture retention, fat melting, and protein denaturation during cooking [7]. For pork, reaching an optimal internal temperature is important to achieve food safety and enhance sensory attributes.

Regardless of cooking temperature degrees, heat is the main influence on the physicochemical properties of pork through the denaturation of proteins and changes within the muscle fibers [6]. According to the United States Department of Agriculture (USDA), it is recommended to cook pork chops to a minimum internal temperature of 63 °C [8]. Cooking pork to lower degrees of doneness has reduced moisture losses and improved eating quality [8]. However, consumers, in most instances, overcook pork products to ensure safety and wholesomeness are achieved [8]. When pork retail cuts are overcooked, it may lead to variation in palatability, whereas cooler degrees of doneness may enhance juiciness or tenderness.

The adoption of newer technologies throughout the meat and food industry, such as the electronic nose, can provide non-destructive informative results on sensory characteristics such as odor and aroma [9,10,11]. Limited research has been conducted on cooked pork chop aromas or odors using non-destructive techniques such as the E-nose. The analytical use of an electronic nose can provide a summary of compositional volatiles and has been used to classify some meat samples, as well as limited efforts to discriminate between fresh and cooked characteristics of meat samples [10]. An electronic nose uses chemical gas sensors in conjunction with flash gas chromatography to identify volatile organic compounds. Previous uses of E-nose technology have identified adulterated meat, provided rapid identification of a food product, and identified pork for halal authentication [12]. Utilizing technologies such as the E-nose may provide greater insight into a meat or food product that would otherwise not be accessible to the industry without conducting extensive quantified laboratory analysis.

Although technology can replace some aspects of a sensory panel, consumer preferences are a key market driver for meat and food products. Therefore, conducting consumer sensory panels is crucial in determining the overall liking and acceptability of a product. Some research concludes that consumers do not regularly use a meat thermometer to determine the degree of doneness of meat but are more likely to if it enhances the flavor or quality of a product [13]. Moreover, the literature concludes that consumers can detect differences in tenderness in pork chops cooked to varying degrees of doneness and varying percentages of phosphate injection [14]. Additionally, recent research shows the influence the degree of doneness has on the color and myoglobin denaturation of beef steaks [15]. However, public acceptance of cooking temperature will require time and education [13].

The previous literature has defined the impact of cooking method, temperature, or degree of doneness on pork and beef quality characteristics [13]. However, there is limited research focusing on the degree of doneness influencing texture, flavor, and consumer acceptability of pork chops. To achieve wholesome pork chop quality and consumer satisfaction, understanding the influence of endpoint cooking temperatures on pork loin chop sensory taste and cooked characteristics is necessary. This study’s objectives were to evaluate the effect of internal endpoint temperatures on the texture, internal cooked color, and consumer acceptability to provide cooking guidelines for improving the quality and safety of pork loin chops.

## 2. Materials and Methods

### 2.1. Raw Materials

Boneless pork loins [USDA Institutional Meat Purchasing Specifications #413] were purchased from a commercial processor and transported to the Lambert–Powell Meat Laboratory at Auburn University. Loins (n = 18) were stored for 14 days from the time of slaughter in refrigeration at 2 °C (Model LEH0630, Larkin, Stone Mountain, GA, USA) until fabrication. Using a bandsaw (Model 334, Biro Manufacturing Co., Marblehead, OH, USA), pork loins were cut parallel to muscle fibers into individual chops (n = 15/loin) at 2.54 cm thick. Chops were vacuum packaged individually in 18.00 cm × 25.40 cm (l × w) 3 mil (nylon/polyethylene) pouches (Prime Source, St. Louis, MO, USA) with an oxygen transmission rate (7.5 cc/100 square inches/24 h of atm) and sealed using a double chamber vacuum packaging machine (Model UV2100-C, Koch Equipment LLC, Kansas City, MO, USA) after being randomly assigned to one of three internal degrees of doneness (63 °C, 71 °C, and 79 °C). Chops were stored frozen (−20 °C ± 1.5 °C) in the absence of light until analysis could be completed.

### 2.2. Cooking Methods

Pork chops were thawed for 12 h at 2.0 °C (±1.5 °C), removed from individual vacuum packaging, and blotted dry with a paper towel (n = 25/treatment). Chops were cooked on aluminum wire rack baking pans in a commercial convection oven (Vulcan, Baltimore, MD, USA) preheated to 176.6 °C. The internal cooking temperatures of each chop from each treatment were monitored by inserting a Teflon-coated thermocouple into the geometric center of each chop attached to a thermometer (Therma K-plus, American Fork, UT, USA). After achieving the required degree of doneness for each treatment, the chops were removed from the oven and cooled to room temperature at 23 °C.

### 2.3. Instrumental Cooked Color Measurement

Once cooked, chops were cooled to 23 °C (n = 10/treatment), sliced horizontally through the geometric center, and scanned for internal cooked color using a HunterLab MiniScan EZ colorimeter (Model 45/0) LAV, Hunter Associates Laboratory Inc., Reston, WV, USA) using illuminant D65 with a 10° observer and an aperture of 31.88 mm. Prior to scanning and again every 30 min, the colorimeter was calibrated using a black and white tile with a repeatability reading of ≤0.05 in accordance with the manufacturer’s guidelines. An average of three readings was taken to determine the internal cooked color of each chop. In addition, the calculated values of hue angle (◦) were determined by [tan^−1^ (b*/a*)], and chroma (C*) was calculated using the following equation: [√ a*^2^ + b*^2^]. Reflectance values from 400 to 700 nm were used to record surface color changes from red to brown using the reflectance ratio of 630 nm/580 nm [16].

### 2.4. Cooking Loss and Texture Analysis

Prior to cooking, chops were weighed on an analytical scale (Model PB2003-2, Mettler Toledo, Columbus, OH, USA) and re-weighed after cooling to room temperature. Cooking loss was calculated using the following formula: [(raw weight − cooked weight)/raw weight × 100].

Texture profile analysis (n = 25/treatment) and Warner–Bratzler shear force (n = 25/treatment) were measured using a texture analyzer (Model TA.XT.Plus 100C, Texture Technologies Corp., New York, NY, USA). After cooking and cooling to room temperature, 1.27 cm diameter cores (n = 6/chop) were removed from each chop. The cores were sheared once using a load cell of 30 kg and a test speed of 2 mm/s.

Texture profile analysis (TPA) was conducted on each pork chop. Three 1 × 1 cm^2^ samples from each pork chop were removed and subjected to a two-cycle compression test with a load cell of 30 kg. Samples were compressed to 50% of the original sample height with a cylindrical probe (TA-25A) 50 mm in diameter with a crosshead speed of 5.00 mm/s. The texture profile analysis parameters were calculated as hardness (kg), which is the force required to compress a sample; cohesiveness, which is sample deformation prior to rupture; springiness, the ability of a sample to return to its original shape after the force is removed; chewiness (kg × cm), the force needed to masticate a sample for swallowing (hardness × cohesiveness × springiness) according to procedures previously described [17,18].

### 2.5. Electronic Nose Testing

Volatile compounds of cooked pork chops (n = 3/treatment) were analyzed by using an electronic nose (Heracles Neo e-nose, Alpha MOS, Toulouse, France) containing an autosampler. Compounds were tested using flash gas chromatography throughout the e-nose evaluation. Under a laboratory fume hood, pork chops from each treatment were trimmed of subcutaneous fat and minced by hand. From the minced sample, 2.00 g of meat from each treatment was weighed and transferred into 20 mL vials. The vials were agitated at 500 rpm with a 50.0 °C incubation temperature for 20 min in the autosampler incubator to generate volatiles for headspace analysis. After incubation, the autosampler injector inserted 5000 mL of the headspace gas at 125 mL/s to concentrate the odor inside the trap. Trapping conditions were maintained at 40.0 °C for 50 s. Hydrogen gas was used at a 1 mL/min flow rate to carry the volatile components into the non-polar (MXT-5) and polar (MXT-1701) capillary columns for chromatographic analysis using a parallel, two-flame ionization detector (FID1 and FID2). Both columns were 180 mm in diameter and 10 m long. The final temperature of the analysis sequence was increased to 250 °C at 1 °C/s temperature increments from the initial 40.0 °C temperature according to described methods [19]. The peaks on the chromatogram were identified by comparing the retention time of each compound with its corresponding retention indices. The detected compounds were further evaluated using a relevance index calculated by ArochemBase Software (Alpha MOS, Toulouse, France, Version 4.7.0). Compounds with a relevance index of 80 or higher were considered for analysis and interpretation.

### 2.6. Consumer Sensory Evaluation

Prior to conducting consumer panel activities, the Auburn University Institutional Review Board (IRB) approved the exempt use of human panelists for this study (Exempt Protocol #Study00000113). Consumer panelists 18 years and older who are regular consumers of pork products were identified and recruited (n = 85). Serving order was determined using RedJade software (version 6.1, Pleasant Hill, CA, USA) so that panelists would evaluate random samples with a masked 3-digit code. Pork chops were cooked to internal temperatures of 63 °C, 71°C, and 79 °C. Pork chops were trimmed free of subcutaneous fat, and with a plastic cutting guide, chops were cut into a 1 × 1 cm^2^ sample from the center of the chop (n = 6/chop) [17]. Samples were presented to panelists randomly in the isolated sensory booths under red-masked lighting with a sample serving temperature of 60 °C. Panelists were instructed to use the saltine crackers (Premium Unsalted Tops, Mondelez Global LLC, East Hanover, NJ, USA) and room temperature water as a palate cleanser between each sample. Consumer panelists evaluated their first impression on a 9-point hedonic scale of “overall liking”, “aroma”, “appearance”, “flavor”, “texture” (1 = dislike extremely; 9 = like extremely), “juiciness” (1 = extremely dry; 9 = extremely juicy), “tenderness” (1 = extremely tough; 9 = extremely tender), and “pork flavor intensity” (1 = none; 9 = extremely intense).

### 2.7. Statistical Analysis

Data were analyzed using the GLIMMIX procedure of SAS (version 9.2; SAS Inst., Cary, NC, USA). Least square means were computed for all dependent variables of internal cooked color, Warner–Bratzler shear force, and texture profile analysis, with treatment serving as the independent variable. Panelists served as a random factor for sensory data analysis. Significant (*p* ≤ 0.05) means were separated using pairwise t-tests (PDIFF option). The analysis of E-nose retention time was calculated using ANOVA means.

## 3. Results and Discussion

### 3.1. Instrumental Cooked Color

Variations in cooked meat color can be attributed to pH, muscle, packaging conditions, fat content, added ingredients, frozen storage, various preservation techniques, and even cooking [20]. Fresh meat characteristics can often alter the cooked and fresh state of color, influencing protein myoglobin, which is responsible for the consumer purchase and acceptability of wholesome meat cuts [21]. Despite previous research and food safety education that encourages consumers to utilize a thermometer when measuring the internal doneness of cooked meat, many consumers simply rely on the cooked color of a product as an indicator of doneness. The current results (Table 1) conclude that pork chops cooked to an internal temperature of 79 °C were darker than chops cooked to 63 °C or 71 °C (*p* < 0.0001). Whereas pork chops cooked to a 63 °C internal temperature were redder (*p* = 0.0057), more yellow (*p* < 0.0001), and more vivid (*p* < 0.0001) internally than chops cooked at greater degrees of doneness. In contrast to the current study, pork chops classified as normal quality using muscle pH and surface color ratings when cooked at higher temperatures were lighter in comparison to those classified as dark, firm, and dry; pale, soft, exudative; or those enhanced with a water-phosphate solution [22]. Surprisingly, there were no differences in hue angle, but as the internal cooking temperature increased, the internal cooked color of the pork chops moved farther from the true red axis (*p* = 0.1209). As expected, upon cooking, the denaturation of myoglobin caused a greater change in red to brown as internal cooking temperatures increased (*p* < 0.0001). Previous results support the current findings indicating that pork chops cooked to a greater internal temperature of 71 °C or 79 °C will be less red than those cooked to an internal temperature of 63 °C [23]. Although internal cooked color is an important factor for the consumer to determine the degree of doneness, previous research supports theories that internal cooked color is not the sole indicator of sensory taste attributes [24]. Additional factors of flavor, such as moisture, texture, and the use of seasonings, can be drivers of consumer acceptability when evaluating meat products.

### 3.2. Cooking Loss and Objective Tenderness

Previous research on consumer acceptability of pork is often focused on tenderness and flavor ratings, which can be directly linked to the moisture lost during cooking procedures [25]. Objective tenderness values of pork loin chops are presented in Table 2. As the degree of doneness for pork chops increased from 71 °C and 79 °C, denaturation and shrinking of the meat muscle structures can occur, often resulting in greater cooking losses (*p* < 0.0001) compared with pork chops cooked to an internal degree of 63 °C [26]. The current results agree with previous findings of cooking loss percentages increasing as the degree of doneness or oven temperatures increased in various pork cuts [26,27]. Consumers often find pork chops cooked to a greater degree of doneness to be less juicy and less tender [28]. It is plausible that the observed cooking loss may be attributed to an increase in objective tenderness of Warner–Bratzler shear force values in the current study. Pork chops cooked to 79 °C exhibited more moisture loss, greater shear force (*p* < 0.0001), hardness (*p* < 0.0001), and chewiness (*p* < 0.0001) values in the texture profile analysis (Table 2). Moreover, research illustrates that as the amount of expelled water increases, the meat becomes less juicy, less tender, and requires more time to chew [29].

### 3.3. Electronic Nose

The mean values for the retention time of volatile compound groups detected by the electronic nose are presented in Table 3. As a result of limited volatile detection in pork chops cooked to varying degrees of doneness, it is plausible that heating meat proteins have a greater influence on flavor volatiles than expected, and additional research is needed for meat cuts, specifically pork. The detection of volatile retention times across all degrees of doneness was not consistent among pork chops, and no significance was calculated. A lack of statistical differences suggests additional research is needed using E-sensors for evaluating flavor components. In cooperation with consumer panelist ratings, the current results indicate that flavor profiles tend to be more likable when the degree of doneness is closer to 63 °C. Electronic sensory instruments are used to distinguish between samples by measuring headspace volatiles of odor or flavor. Cooking temperature can influence the volatile profile because of Maillard and lipid reactions that occur in meat products [30,31]. Previous research on volatile compound profiles of cooked pork cheeks suggests that cooking to a higher degree of doneness will have a positive influence on compounds associated with the Maillard reaction [32]. The chemical composition of cooked meat, such as alcohols, can be influential to the overall aroma of cooked meat [32]. In the current study, alcohols were detected in pork chops cooked at 63 °C and 79 °C but were not detectable in chops cooked at 71 °C. It is plausible that the degradation of alcohols at intermediate cooking temperatures or an interaction with other compounds during cooking likely occurred [32]. Aldehydes often contribute to meaty and fatty aromas and were present in cooked chops linked to lipid oxidation that is reported at greater cooking temperatures [30]. Carboxylic acids are associated with sour and rancid notes and were only detected at 71 °C. Previous literature reports indicate this could be due to a peak in lipid oxidation and the breakdown of triglycerides [31]. The results of the current study align with previous research emphasizing the role of temperature on the flavor and aroma profiles of a product [32,33].

### 3.4. Consumer Panel

Consumer panelists (Table 4) evaluated the sensory attributes of pork chops to identify the influence of internal cooking temperature. The degree of doneness of a pork product can impact the eating experience more than visual characteristics [20]. The results from the current study support previous theories that the degree of doneness can significantly influence consumer panelist sensory ratings of pork chops to be juicier and more tender when cooked to a medium degree of doneness (Table 5). Pork chops were rated juicier when cooked at 63 °C compared with those chops cooked at 71 °C or 79 °C (*p* < 0.0001). Similarly, sensory tenderness ratings were greater in pork chops cooked at 63 °C, compared with chops cooked at 71° or 79° (*p* < 0.0001). The consumers detected no change in the pork flavor intensity between the three temperatures (*p* = 0.6578). Although there was no difference identified in the overall liking (*p* = 0.0649), the results show a tendency for participants to prefer pork chops cooked at 63 °C compared with 71 °C or 79 °C. In addition, there was no difference in the overall aroma (*p* = 0.6825) or overall flavor (*p* = 0.1228) of the pork chops (Table 6). Furthermore, studies using trained sensory panelists have concluded significant differences can be detected for the sensory anchor juiciness and overall tenderness of pork chops when cooked at 63 °C, 68 °C, 74 °C and 79 °C [34]. In agreement with the current study, pork chops cooked to 63 °C have been rated juicier and more tender [34]. Endpoint cooking temperature can influence the consumer’s eating experience, but consumer education on the degree of doneness is needed. However, hesitation by the consumer to alter the cooking of pork chops to revised cooking temperatures will require greater time and more cooking education [35].

**Table 4:** Consumer sensory panel frequency demographics.

	Percentage
**Age**	
18 to 20 years	2
21 to 29 years	68
30 to 39 years	19
40 to 49 years	8
50 to 59 years	3
**Gender**	
Male	44.2
Female	55.8
**Highest Level of Education**	
Graduate degree	52.8
4-year college degree	34.4
2-year college degree	2.8
High school diploma or GED	10
Less than high school	0
**Ethnicity**	
Caucasian	34.8
Latino/Hispanic	40.3
Asian or Pacific Islander	10
African American	10.9
Prefer not to respond	4
Other	0
**Income**	
Less than $30,000	74.2
$30,000 to $49,999	7.2
$50,000 to $70,999	10
Greater than $80,000	8.6
**Consume Pork Products**	
Once a day or more	1.4
More than 3 times per week	6.3
2 to 3 times per week	27.2
Once a week	19.7
2 to 3 times per month	24.9
Once a month	15.8
Less than once a month	4.7
**Purchase Pork Products**	
Once a week	12.2
Once every 2 or 3 weeks	26.2
Once a month	40.5
Once every 2 or 3 months	8.9
Once every 4 to 6 months	6.3
Once or twice a year	7.2
Less than once a year	1.4

**Table 5:** Consumer panelist intensity ratings of pork chops at various cooking endpoint temperatures.

Treatment					
Sensory Anchor ^1^	63 °C	71 °C	79 °C	SEM *	*p* Value
**Juicy**	6.80 ^a^	5.62 ^b^	4.04 ^c^	0.208	<0.0001
**Tenderness**	6.47 ^a^	5.74 ^b^	4.88 ^c^	0.199	<0.0001
**Pork Flavor Intensity**	5.71	5.76	5.24	0.181	0.6578

^1^ Sensory anchors for consumer panelist ratings represent the use of a 9-point hedonic scale for juicy (1 = extremely dry; 9 = extremely juicy); tenderness (1 = extremely tough; 9 = extremely tender); and pork flavor intensity (1 = none; 9 = extremely intense). ^a–c^ Mean values within a row lacking common superscripts differ (*p* < 0.05). * SEM, standard error of the mean.

**Table 6:** Consumer panelists’ overall ratings of pork chops at various cooking endpoint temperatures.

Treatment					
Sensory Anchor ^1^	63 °C	71 °C	79 °C	SEM *	*p* Value
**Overall Liking**	6.14	6.09	5.65	0.231	0.0649
**Overall Appearance**	6.62	6.40	6.24	0.179	0.0915
**Overall Aroma**	6.36	6.21	6.18	0.186	0.6825
**Overall Flavor**	6.32	6.32	5.87	0.213	0.1228
**Overall Texture**	6.65 ^a^	6.14 ^a^	5.27 ^b^	0.273	<0.0001

^1^ Sensory anchors for consumer panelist ratings represent the use of a 9-point hedonic scale (1 = dislike extremely to 9 = like extremely); ^a–b^ Mean values within a row lacking common superscripts differ (*p* < 0.05). * SEM, standard error of the mean.

## 4. Conclusions

Conducting research on the optimal degree of doneness temperature for pork is important for maintaining production practices that will culminate in high-quality and satisfactory pork products for consumers. The current results determined that cooking pork chops to a lower degree of doneness improved moisture retention, resulting in an improved flavor profile and objective tenderness values. Ultimately, this research supports cooking pork to endpoint temperatures similar to other red meats such as beef and lamb, which will result in a more tender, less chewy, and improved texture for pork loin chops. These changes in cooking were caused by variations in the degree of doneness and improved consumer ratings for overall texture, a juicier pork chop, and improved tenderness, which are the most important flavor attributes in guiding consumer satisfaction. Adopting the recommended endpoint temperature of 63 °C could improve consumer satisfaction by delivering a pork loin chop that is preferred by consumers.

## Figures and Tables

**Table 1:** Instrumental cooked color of pork chops cooked to various endpoint temperatures.

	Treatment				
	63 °C	71 °C	79 °C	SEM *	*p* Value
Lightness (L*) **^1^**	76.80 ^a^	76.17 ^b^	73.59 ^c^	0.255	<0.0001
Redness (a*) ^2^	3.52 ^a^	2.96 ^b^	2.95 ^b^	0.204	0.0057
Yellowness (b*) ^3^	17.41 ^a^	16.07 ^b^	15.35 ^b^	0.451	<0.0001
**Hue Angle (H°) ^4^**	78.62	79.62	79.32	0.531	0.1209
Chroma (C*) ^5^	17.77 ^a^	16.36 ^b^	15.64 ^b^	0.466	<0.0001
**Red to Brown ^6^**	1.58 ^a^	1.29 ^b^	1.21 ^b^	0.047	<0.0001

^1^ Lightness (L*)–values are a measure of darkness to lightness (a larger value indicates a lighter color); ^2^ Redness (a*)–values are a measure of redness (a larger value indicates a redder color); and ^3^ Yellowness (b*)–values are a measure of yellowness (a larger value indicates a more yellow color). ^4^ Hue angle (H°) represents the change in color from the true red axis (a larger number indicates a greater shift from red to yellow). ^5^ C* (Chroma) is a measure of total color (a larger number indicates a more vivid color). ^6^ Red to brown is the reflectance ratio of 630 nm ÷ 580 nm and represents a change in color from red to brown (a larger value indicates a redder color). ^a–c^ Mean values within a row lacking common superscripts differ (*p* < 0.05). * SEM, standard error of the mean.

**Table 2:** Cooking loss and objective tenderness values for pork chops at various endpoint temperatures.

	Treatment				
	63 °C	71 °C	79 °C	SEM *	*p* Value
**Cooking Loss (%)**	19.81 ^c^	30.37 ^b^	39.65 ^a^	1.041	<0.0001
**WBSF (N)**	17.40 ^c^	21.26 ^b^	23.83 ^a^	0.931	<0.0001
**Hardness (kg)**	5.45 ^c^	7.64 ^b^	9.09 ^a^	0.313	<0.0001
**Springiness ^1^**	0.53 ^b^	0.55 ^b^	0.61 ^a^	0.009	<0.0001
**Cohesiveness ^2^**	0.54 ^a^	0.52 ^a^	0.49 ^b^	0.009	0.0060
**Chewiness ^3^**	1.59 ^c^	2.22 ^b^	2.74 ^a^	0.141	<0.0001
**Resilience ^4^**	0.24 ^a^	0.23 ^b^	0.19 ^c^	0.004	<0.0001

^1^ Values for springiness (ratio of the time duration of force input during the second compression to that during the first compression or length 2/length 1). ^2^ Values for cohesiveness (ratio of the positive force area during the second compression to that during the first compression, area 2/area 1). ^3^ Chewiness = hardness × cohesiveness × springiness. ^4^ Values for resilience (ratio of the time duration of force input during the first compression or area 5/area 4) ^a–c^ Mean values within a row lacking common superscripts differ (*p* < 0.05). * SEM standard error of the mean.

**Table 3:** Mean retention time of electronic nose volatile compound groups in pork chops cooked to various endpoint temperatures.

	Treatment			
Compound Group	63 °C	71 °C	79 °C	Sensory Descriptors
**Alcohols**	67.61	ND ^1^	64.22	Pleasant; Burnt
**Aldehydes**	ND ^1^	35.79	21.81	Pungent
**Alkanes**	46.90	56.47	ND ^1^	Meaty
**Carboxylic Acids**	ND ^1^	30.80	ND ^1^	Sour; Rancid
**Halogenated Compounds**	37.24	26.51	47.99	Off-odor
**Lactones**	ND ^1^	79.38	83.85	Fatty
**Others**	22.29	22.28	56.64	Sweet; Pungent

^1^ ND (not detected within treatment).

## Data Availability

The original contributions presented in this study are included in this article; further inquiries can be directed to the corresponding author.

## Abbreviations

The following abbreviations are used in this manuscript:

USDA	United States Department of Agriculture
TPA	Texture profile analysis
IRB	Institutional Review Board
WBSF	Warner–Bratzler shear force
ND	Not detected
SEM	Standard error of the mean
